# Sex Determination in *Dioscorea dumetorum*: Evidence of Heteromorphic Sex Chromosomes and Sex-Linked NORs

**DOI:** 10.3390/plants12020228

**Published:** 2023-01-04

**Authors:** Florence Ngo Ngwe, Sonja Siljak-Yakovlev

**Affiliations:** 1 Biodiversity Division, Institute of Agricultural Research for Development, Yaoundé 2123, Cameroon; 2 Université Paris-Saclay, CNRS, AgroParisTech, Ecologie Systématique Evolution, 91190 Gif-sur-Yvette, France

**Keywords:** dioecy, GC-rich DNA region, heterochromatin, molecular cytogenetics, rDNA, sex chromosomes, sex-linked NORs

## Abstract

Yams (*Dioscorea* spp.) are a pantropical genus located worldwide that constitute an important source of nutrients and pharmaceutical substances. Some *Dioscorea* crop species are widely grown in West Africa. One species that is mainly cultivated in Cameroon is *Dioscorea dumetorum.* This is a dioecious root crop whose sex-determining system was unknown until now. To address the possible presence of sex chromosomes in *D. dumetorum,* we performed a karyotype characterization of male and female individuals using classical and molecular cytogenetic approaches. It was determined that 2*n* = 40 was the most common number of chromosomes in all of the investigated samples. One chromosome pair was longer than the others in the chromosome set and was a heteromorph in male and homomorph in female individuals. This pair corresponded to sex chromosomes, and we also confirmed this with molecular cytogenetic experiments. The results of chromomycin banding revealed the presence of strong positive signals on this chromosome pair. The signals, corresponding to GC-rich DNA regions, were similar in size on the chromosomes of the female individuals, whereas they were different in size in the male individuals. This size difference in the GC-rich heterochromatin regions was also apparent in the interphase nuclei as one small and one large fluorescent spot. The results of the in situ hybridization experiment showed that these chromomycin positive signals on the sex chromosomes also corresponded to the 35S rDNA cluster. The mean 2C DNA value (genome size) obtained for *D. dumentorum* was 0.71 pg (±0.012), which represents a small genome size. We found no difference in the genome size between the male and female individuals. The results of this study contribute to increasing our knowledge of sex determination in *D. dumetorum* (standard sex-determining XX/XY system) and may have some agronomic applications.

## 1. Introduction

The pantropical genus *Dioscorea* Plum. ex L. (yam) comprises approximately 600 species that are mainly widespread in tropical regions [1,2] and includes a large number of important tuber crops. These species are mostly dioecious (separate male and female individuals) and belong to the family Dioscoreaceae R.Br. Yams are a staple food for Central and West African populations, and they increase the food security in this region of the continent [3]. The top seven yam producers in the world are Nigeria, Ivory Coast, Ghana, Benin, Togo, Central African Republic, and Cameroon [4]. In total, these countries produce 11 of the 600 yam species found around the world: *D. alata* L., *D. esculenta* (Lour.) Burkill, *D. oppositifolia* L., *D. japonica* Thunb., *D. nummularia* Lam., *D. pentaphylla* L., *D. rotundata* Poir. [synonym of *Dioscorea cayennensis* subsp. *rotundata* (Poir.) J. Miège], *D. dumetorum* (Kunth) Pax, *D. cayennensis* Lam., *D. trifida* L.f, and *D. bulbifera* L. [5]. Among these yam species, *D. alata*, *D. cayennensis*, *D. dumetorum*, and *D. rotundata* are the species that are cultivated in all of the agroecological zones of Cameroon (which is a very important fact in the context of climate change), and their use increases the strength of the economy in this country [6]. *Dioscorea dumetorum* has the highest nutritional value of all species of the genus because its tubers are very rich in protein (9.6%). In addition, they contain a starch that is easily digestible due to its crystal structure that is close to that of cereal starch [7,8].

*Dioscorea dumetorum*, known as the bitter yam, is easily distinguished from others species of the genus by the morphology of its trifoliate, pubescent, thick leaves, and loop shape tubers [9,10]. In addition to nutrition, this species is also used for the medicinal purposes, namely for the treatment of diabetes [11]. The tubers contain dioscoretine (water soluble glycoside), which is exploited in pharmaceutical industries as a hypoglycemic agent [11].

In spite of its multiple uses, yam production is globally faced with several constraints, mainly the lack of high quality seeds. The main constraint, common to all cultivated yam species, is the farmers’ practice of vegetative reproduction. In general, farmers use tubers from previous harvests for the new plantation. This type of vegetative reproduction is responsible for a yield reduction of approximately 80% [12] and also for a dramatic loss of genetic diversity. In addition to these constraints, another problem was observed, especially regarding *D. dumetorum*. The postharvest hardening of tubers occurs two or three days after harvest, which renders the tubers inedible. Therefore, such tubers cannot be used for human consumption. Thus, *D. dumetorum* tubers should be boiled immediately after harvest and marketed in cooked form [13].

Numerous studies have conducted to characterize the genome and its diversity, and to also increase *D. dumetorum* productivity [14,15,16,17,18,19,20]. Yolou et al. [21] recommended the use of sexual reproduction to increase the quality of *D. dumetorum* because their cultivars, similar to those of the *D. alata* and *D. cayenensis-rotundata* complex, produce viable seeds. Researchers have made considerable progress in genetic improvement by sexual hybridization in *D. rotundata* [22] and *D. alata* [23] via sexual hybridization. In the latter species, polyploidy and infertility were related, as polyploid females were sterile [24]. Knowledge of the genome and breeding system is the key to any successful enhancement program for yams, especially considering that they are a dioecious species.

Although the dioecy is present in ~40% of angiosperm families, it is rare in the final analysis [25]. In contrast to gymnosperms, which present even 65% of dioecious species [26] in angiosperms, only approximately 6% or 14,600 species from 960 genera and 200 families are dioecious [27,28]. Ming et al. [29] estimated the occurrence of dioecious species among land plants at 10% and highlight the fact that dioecy presents the precondition for sex chromosome evolution. Curiously, dioecy was recorded in only three monocot families (*Phoenix*, *Asparagus*, and *Dioscorea*) [30]. They noted that the genus *Dioscorea* would not be appropriate subjects for evolutionary and phylogenetic studies of sex determination because it includes too many species. They stressed, however, that “it was important to study sexual dimorphism in cultured species of the genus and finally to demonstrate or not the existence of sex chromosomes”. This was the main purpose of this study.

Montalvão et al. [31] emphasized the diversity of sex-determining systems in dioecious species and the importance of studying them especially for crop plants, which could lead to an increase in their agricultural value.

Genome size measurement and karyotype analyses should be used to characterize the genome of dioecious species. Genome size is important for basic and applied studies involving germplasm enhancement [32]. Some authors have also mentioned cytological and chromosomal markers as essential tools for any genetic selection program [33,34]. These markers have been used, for example, to observe the sex chromosomes of dioecious plants such as the widely cultivated date palm [35].

Karyological studies of *D. dumetorum* are scarce and, to our knowledge, no studies using molecular cytogenetics have been reported for this species or for the genus *Dioscorea* in general. The fact that the dioecious species of the genus, especially those that are cultivated, have not attracted more interest on the karyological and cytogenetic level is strange.

With the present work, we mainly aim to more thoroughly understand the sex-determining system of Dioscorea *dumetorum.* To achieve this, we first investigated a possible presence of sex chromosomes via a karyotype characterization of male and female individuals using conventional and molecular cytogenetic approaches: fluorochrome banding to detect GC-rich DNA regions and Fluorescent In Situ Hybridization (FISH) to physically map rRNA genes on chromosomes. In addition, to check an eventual difference in DNA amount between male and female individuals, we evaluated the genome size with flow cytometry.

## 2. Results

### 2.1. Genome Size and Chromosome Number

The mean 2C DNA content obtained for two accessions of *D. dumentorum* (individuals in these samples were mixed males and females) from Southwest and Central Cameroon was 0.716 pg (±0.012; CV% = 1.64) and 0.716 (±0.718; CV% = 1.99), respectively. No significant difference was found in the genome size between male and female individuals assessed separately in three accessions (Table 1).

**Figure 1:**
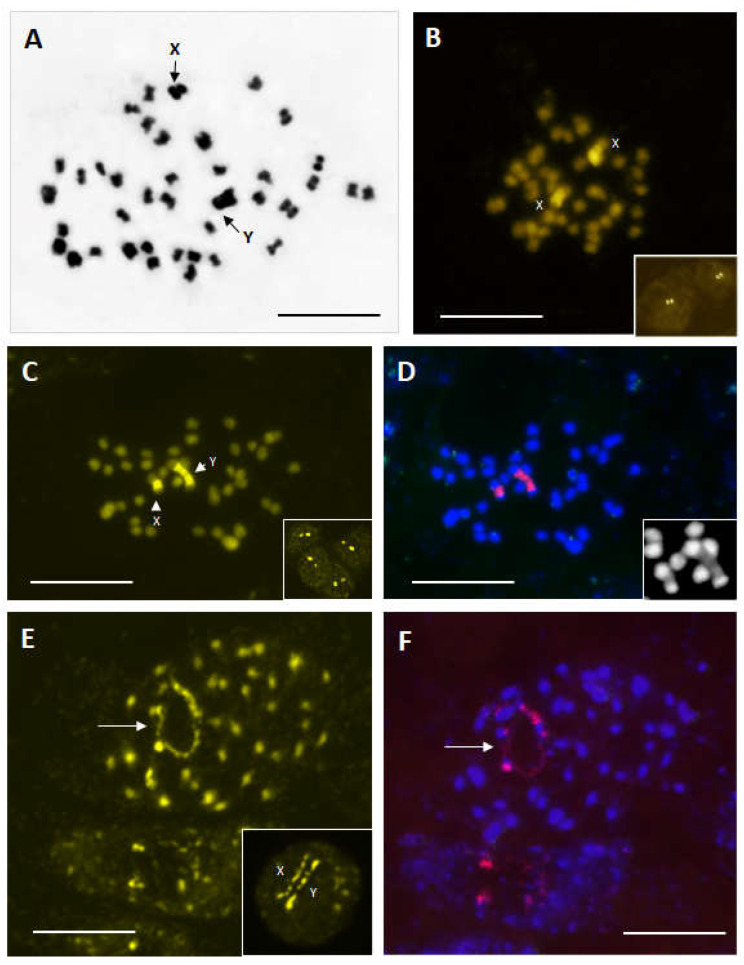
(**A**–**F**) orcein-stained metaphase of male individual (2*n* = 40) with sexual chromosomes X and Y indicated by arrows (**A**). Chromomycin-stained metaphase and interphase nuclei (framed details) of female (**B**) and male (**C**) individuals. Note the strong difference in size of CMA signals in male individual (**C**). Same chromosome plate after FISH experiment. CMA signals were also strongly labelled with rDNA 35S probe (**D**, red signals) but were DAPI negatives (**D**, framed detail). Same prometaphase plate chowing activity of rRNA genes (arrows) stained by chromomycin (**E**) and after FISH (**F**). The beginning of decondensation of sex chromosomes at the early prophase (**E**, framed detail). Bar = 10 µm.

After Feulgen staining, we found that 2*n* = 40 was the most frequent chromosome number inl the investigated samples. In some cells, the numbers of 2*n* = 36 or 38 were rarely observed. Two heteromorphic chromosomes, that were longer than the other chromosomes in the set, were observed in all male individuals (Figure 1A, arrows). In the female individuals, one chromosomal pair was also larger than the other chromosomes in the karyotype, but it was homomorphic compared with that of the male individuals. These chromosomes corresponded to sex chromosomes (XX/XY), which was additionally confirmed with fluorochrome banding (chromomycin—CMA) and FISH (Figure 1B–F).

### 2.2. Heterochromatin and rDNA Physical Mapping

The chromomycin banding revealed that the biggest chromosome pair observed after Feulgen staining presented strong CMA^+^ bands. These bands (signals) corresponded to heterochromatinized GC-rich DNA regions. In the female individuals similar size on the chromosomes (Figure 1B) and in the interphase nuclei (Figure 1B, framed detail). In contrast, such signals were a different size in the chromosomes and interphase nuclei of the male individuals (Figure 1C, arrows, and framed detail).

The FISH experiment confirmed this observation and showed that the CMA^+^ DNA regions corresponded to 35S rDNA sites, strongly labelled with the 18S-5.8S-26S probe (Figure 1D, red signals). These GC-rich heterochromatin normally appeared to be DAPI (4’,6-diamidino-2-phenylindole) negative (AT-poor regions) when observed only with the DAPI filter (Figure 1D, framed detail).

The activity of both 35S sites was also demonstrated after CMA fluorochrome banding and FISH (Figure 1E and Figure 1F, respectively).

However, an uneven chromatin decondensation level of sex chromosomes in the male individuals was noticed. The X chromosome decondensed more than the Y chromosome for which the terminal heterochromatin blocks that remained highly condensed were well visible (Figure 1E, framed detail).

From all these results, we can conclude that a standard sex-determining XX/XY system is to the basis of sex determinism in *D. dumetorum*.

## 3. Discussion

### 3.1. Chromosome Feature and Genome Size

For several decades, karyological studies have been conducted for the genus *Dioscorea* [36,37,38,39,40,41,42,43] and reported chromosome numbers ranged from 2*n* = 18 to 2*n* = 144, which demonstrates a varying ploidy level (from 2*x* to 13*x*). The majority of *Dioscorea* species are polyploid, but the tetraploid cytotype (2*n* = 4*x* = 40) is becoming the more common [42,44].

The results of karyological analyses of the *Dioscorea* genus demonstrated the existence of three base chromosome numbers (*x* = 9, 10, and 12). The basic chromosome number of *x*= 9 was mainly reported in *Dioscorea* species from the New-Word [39,42,45] whereas *x* = 10 is mainly found in African [37,39,42] and Asian representatives of the genus [46,47,48]. The basic number of *x* = 12 was found in two European paleoendemic diploid species: *D. pyrenaica* Bubani & Bordère ex Gren. and *D. balcanica* Košanin [39]. The results of all these studies show that the descending disploidy (from *x* = 12 to *x* = 9) and polyloidy (from 2*x* to 8*x* for *x* = 10 and from 2*x* to 16*x* for *x* = 9) are the main evolutionary mechanisms in the genus *Dioscorea*.

We found that *D. dumetorum* from Cameroon displayed a stable chromosome number 2*n* = 40, which confirmed the basic chromosome number of *x* = 10 and a tetraploid state of this cytotypes with a secondary basic chromosome number of *x* = 20. Miège [37] and Baquar [40] already reported the same number of chromosomes for *D. dumetorum*. Miège [38] and Martin and Ortiz [39] found 2*n* = 36, 45, and 54 for *D. dumetorum,* and therefore demonstrated the existence of variability in the chromosome number and ploidy level of this species. In some *Dioscorea* species, several ploidy levels exist: in *D. alata,* the authors of [49,50] reported tetraploid and octoploid chromosome numbers, respectively, and in a *D. cayenensis-rotundata* complex, the authors of [51] even detected three ploidy levels (4*x*, 6*x,* and 8*x*).

Several *Dioscorea* species*,* which are considered tetraploid, have been revealed as diploid by the microsatellite markers, namely a diploidized tetraploid, with the secondary basic chromosome number *x* = 20. It is the case of *D. rotundata* [52] and *D. alata* [53]. The similar studies, coupled with a meiosis study, should be performed on *D. dumetorum* to confirm its basic chromosomic number and consequently its ploidy state, which is important for genetic diversity studies and breeding programs.

The genome size of *Dioscorea* species with 2*n* = 40 range from 0.70 pg in *D. dumentorum* to 2.10 pg in *D. esculenta* [20,51,54], which presents a large panel of variation for the same ploidy level. *Dioscorea dumentorum* with 2C values of 0.71 pg belong to the very small genome category (2C ≤ 2.8 pg) according to the categories established by Leitch et al. [55]. A similar value was already reported for *D. dumetorum* from Benin, Nigeria, Ghana, Togo [54], and Cameroon [16]. In this study, we did not detect any significant difference in genome size between male and female individuals.

Because *D. dumetorum* has a very small genome (0.71 pg/2C or 347 Mbp/1C in the present data; 322 to 350 Mbp/1C reported by Siadjeu et al. [20]), this species could be a useful model to study sex chromosome evolution in the genus *Dioscorea,* of which almost all the species are dioecious.

### 3.2. Molecular Cytogenetics Characterization of Sex Chromosomes

The results of the cytological analyses after Feulgen staining revealed two heteromorphic chromosomes that were longer than the other chromosomes in the male individuals. These chromosomes corresponded to sex chromosomes, which was additionally confirmed by fluorochrome banding and FISH methods. The use of these techniques allowed us to solve the difficulties concerning the cytogenetical studies of the genus *Dioscorea*.

In the past, several studies have been performed to show a possible presence of sex chromosomes in the *Dioscorea* genus via cytological observations. Some reports suggested the presence of heteromorphic sex chromosomes [36,56,57], whereas others did not [39,47]. However, none of these authors were able to cytologically demonstrate the presence of sex chromosomes. Given the difficulties associated with observing chromosomes, several authors have used genetic or molecular methods as an alternative to validate the presence of sex chromosomes in the *Dioscorea* genus [20,58,59,60,61,62]. Researchers typically used *Dioscorea tokoro* as a model species for genetic studies because creating a controlled cross of *D. tokoro* is relatively easy [59,63]. These genetic studies led scientists to propose that the male, but not the female, determines the sex of the progeny, and that the male is the heterogametic. Following the sequencing of the *D. dumetorum* genome, Siadjeu et al. [20] suggested “that only one locus may be involved in sex determination and may be inherited via a system of XX/XY sex chromosomes involving heterogametic males (XY)”.

We initiated these studies as part of a thesis [64] and presented the first results at a conference in 2019 [65]. We implemented chromomycin banding and FISH techniques, which made it possible to demonstrate the presence of sex chromosomes and characterize them in the *D. dumetorum* karyotype. These chromosomes are homomorphic in female (XX) individuals whereas they are heteromorphic in male (XY) individuals. The X and Y chromosomes are easily distinguished by their size, as the Y is the largest, and by the size of the CMA and FISH signals. The strong signals corresponding to GC-rich heterochromatin could be explained by the accumulation of repetitive sequences due to the lack of recombination between the sex chromosomes.

The application of the FISH protocol to other dioecious species such as the palm date also revealed differences in sex chromosome structures [35]. The sex chromosome structure found in *D. dumetorum* with both chromomycin banding and physical rDNA mapping was similar to those of the corresponding chromosomes present in the palm date [35].

Approximately 40 species (from 21 genera and 15 families of angiosperms) are known to have sex chromosomes, and of these, only ~20 species from six genera (*Cannabis*, *Humulus*, *Silene*, *Coccinia*, *Trichosanthes*, and *Rumex*) have heteromorphic sex chromosomes [29]. Now, because of the results we obtained in this study (the cytologically observable heteromorphic sex chromosomes), the genus *Dioscorea* can be added to this list.

### 3.3. Unusual Association of rDNA Cluster and NORs with Sex Chromosomes

Another finding worth highlighting is that *D. dumetorum* sex chromosomes also carry ribosomal genes (in the NORs), which have already reported in date palms and some other plant species such as *Spinacia oleracea* [66] and *Marchantia polymorpha* [67]. Therefore, these chromosomes are of particular importance in the genome as they carry both the sex-determining region and the ribosomal genes that control nucleolus formation. This phenomenon has also been detected in animals, especially in fish [68,69] and very rarely in mammals [70].

Charlesworth [71] stressed that the sex chromosome systems independently arose several times in angiosperms from hermaphroditic ancestors (mainly monoecious species). According Hobza et al. [72,73], “an early event shaping the Y chromosome” could be a consequence of a recombination restriction caused by the accumulation of repetitive sequences particularly on the Y chromosomes. In our case, these sequences were particularly present in the rDNA cluster region, which was considerably larger on Y chromosomes than on X chromosomes. The fact that rDNA was presently found in sex chromosomes (sex-linked NORs) may suggest that the origin of these chromosomes is related to the nucleolar autosomes of hermaphroditic ancestors.

## 4. Materials and Methods

### 4.1. Plant Materials

The materials included yam tubers from male and female *D. dumetorum* individuals that we collected in the central and southwest regions of Cameroon (Table 1). These regions belong to the agro-ecological zones where yam production is the most important. The tubers were enveloped in blotting paper and placed in plastic bags to initiate root development. The first roots appear after two weeks and the first leaves after three to four weeks. The secondary root tips obtained after germination were used for the cytogenetic studies and the first leaves were used for the genome size assessment.

### 4.2. Chromosome Number Determination

We pretreated secondary roots tips, obtained from the germination of the tubers in the laboratory, with 0.002 M 8-hydroxyquinoline at 16 °C for 3 h and performed a fixation in freshly prepared 3:1 (*v/v*) ethanol–acetic acid for at least 48 h. After this first fixation, the root tips could be stored in ethanol 70% at −20 °C for several months. We hydrolyzed the roots tips in 1N HCL for 16 min at 60 °C and stained them with a Schiff reagent using the standard Feulgen method. To intensify the staining, we performed the squash in a drop of orcein or acetic carmine and determined the chromosome number on several well-spread metaphase plates from at least 5 individuals per accession.

### 4.3. Genome Size Assessment Using Flow Cytometry

The leaves of *D. dumentorum* were collected from tubers sprouted in germination at the laboratory. For each accession, nuclei were isolated following the method of Bourge et al. [74]: 50 mg of yam leaves together with 20 mg of leaves from the internal standard [tomato, *Solanum lycopersicum*, 2C = 1.99 pg [75] were co-chopped with a razor blade in a Petri dish containing 1 mL of cold Gif nuclear isolating buffer—GNB (Bourge et al. 2018): 45 mM MgCl_2_, 30 mM Sodium-Citrate and 60 mM MOPS acid pH 7.0, 1% PVP 10.000, RNAse (2.5 U/mL) and 10 mM sodium metabisulfite (S_2_O_5_.Na_2_), which is a reducing agent that is less toxic than β-mercaptoethanol. This GNB buffer can be complemented by 0.5% Triton X-100. The nuclei suspension was filtered through nylon mesh (pore size 30 μg/mL; Sigma, Ronkonkoma, NY, USA) and nuclei were stained with 100 μg/mL propidium iodide (PI), a specific DNA fluorochrome intercalating dye, and kept it for 5 min at 4 °C.

Ten or five individuals from two accessions were analyzed for average genome size. Two individuals, one male and one female, from three accessions were measured to assess the possible difference between the two sexes. At least 5000 nuclei were measured using a Partec CyFlowSL3 (Partec, Münster, Germany) 532-nm laser cytometer. Fluorescence histograms were analyzed for each sample and internal standard. The nuclear DNA content was estimated using the linear relationship between the fluorescent signals from stained nuclei of the *Dioscorea* specimens and the internal standard obtained with the following formula:

2C DNA content/nucleus (pg) = (Yam 2C peak mean/Tomato 2C peak mean) × Tomato 2C DNA (pg)

### 4.4. Fluorochrome Banding and Fluorescence In Situ Hybridization

#### 4.4.1. Protoplast Preparation

The fixed root tips were first washed in a 0.01 M citrate buffer (pH 4.6) for 15 min, then incubated in an enzymatic mixture composed of 4% cellulase RS (Onozuka Yakult Honsha Co., Tokyo, Japan), 1% pectolyase Y23 (Seishin Pharmaceuical Co, Tokio, Japan), and 4% hemicellulase (Sigma Chemical Co., Ronkonkoma, NY, USA) in a 0.01 M citrate buffer at pH 4.6. Enzymatic digestion time depended on the size of the roots and was carried out from 30 min to 1 h at 37 °C. Then, the digested meristems were squashed onto a drop of freshly prepared 50% acetic acid and the preparations were observed with a phase contrast microscope. The best slides were frozen at −80 °C for at least 12 h. The cover slips were removed and the slides were rinsed with absolute ethanol and air-dried.

#### 4.4.2. Fluorochrome Banding

The chromomycin A_3_ (CMA) fluorochrome was used for detection of the GC-rich DNA regions. The technique of fluorochrome banding was performed, following Schweizer [76] with minor modifications from Siljak-Yakovlev et al. [77]: chromomycin concentration was 0.2 mg/mL, staining time was 60 min and slides were mounted in citifluor AF2 (Agar Scientific Ltd., Stanstead, Essex, UK). Some slides were used for the FISH experiment after discoloration in an ethanol-glacial acetic acid solution (3/1, *v/v*), and dehydration in a graded ethanol series (70%, 90%, 100%) for 3 min each and then air-drying.

#### 4.4.3. Florescence In Situ Hybridization

The fluorescence in situ hybridization (FISH) was performed following Heslop-Harrison et al. [78] with slight modifications. The FISH was accomplished to detect the 35S rRNA genes. The 35S rDNA probe was a clone of 4-kb from the *Eco*RI fragment, which included 18S-5.8S-26S rDNA sequences from *Arabidopsis thaliana* (L.) Heynh labelled with the direct Cy3 fluorochrome (Amersham, Courtaboeuf, Les Ulis, France) by a nick translation, according to the manufacturer’s protocol.

Observation of the chromosome plates was made with an epifluorescence Zeiss Axiophot microscope with different combinations of excitation and emission filter sets (01, 07, 15) and the triple filter (25). Hybridization signals were analyzed using a highly sensitive CCD camera (Retiga 2000R; Princeton instruments, Evry, France). The images of the 35S rDNA patterns were overlapped using a Metavue Image Analyzer (Evry, France) to obtain a final FISH image.

## 5. Conclusions

Sex determination in *D. dumetorum* is based on the standard XX/XY sex determining system. We confirmed the presence of heteromophic sex chromosomes in male individuals and homomorphic sex chromosomes in female individuals with classic and molecular cytogenetic approaches. These chromosomes were the only ones in the chromosomal set to have strong heterochromatic GC-rich DNA regions of unequal size in the male individuals. The ribosomal genes (rRNA) or sex-linked NORs were located in the same regions. The sex-determining genes are also probably located in this non-recombining region. Because *D. dumetorum* has a very small genome (347 Mbp/1C), the sex-determining regions may be identified in the near future with genome sequencing, which will be carried out in our future research. The results presented in this study contribute to enhancing our understanding of *D. dumetorum* sex determination and opens new perspectives for the improvement and resumption of interspecific crossing and selection programs in yam crops.

## Figures and Tables

**Table 1:** Origin of material and 2C DNA values obtained for *Dioscorea* studied accessions.

	Collection Site	Geographic Coordinats Latitude (N) Longitude E	2C DNA in pg (SD; CV%)	N° of Measured Individuals
*D. dumetorum* mix *	Muyaka	4°43′18″ N 9°38′27″ E	0.714 (0.012; 1.64)	5
*D. dumetorum* mix *	Mbangassina	4°37′47″ N 11°42′25″ E	0.718 (0.014; 1.99)	10
*D. dumetorum* ♂	Ombessa	4°36′0″ N 11°15′0″ E	0.712	1
*D. dumetorum* ♀	Ombessa	″ ″	0.720	1
*D. dumetorum* ♂	Ekona	4°14′00″ N 9°20′04.0″ E	0.694	1
*D. dumetorum* ♀	Ekona	″ ″	0.714	1
*D. dumetorum* ♂	Bafoussam	5°28′66″ N 10°25′03″ E	0.729	1
*D. dumetorum* ♀	Bafoussam	″ ″	0.718	1

* The individuals in these samples were mixed, males and females.

## Data Availability

Not applicable.

## References

[1] Knuth R., Engler A. (1924). Dioscoreaceae. Das Pflanzenreich.

[2] Viruel J., Segarra-Moragues J.G., Raz L., Forest F., Wilkin P., Sanmartín I., Catalán P. (2016). Late cretaceous-early eocene origin of yams (*Dioscorea*, Dioscoreaceae) in the Laurasian Palaearctic and their subsequent Oligocene-Miocene diversification. J. Biogeogr..

[3] Bricas N., Attaie H. (1998). La consommation alimentaire des ignames. Synthèse des connaissances et enjeux par la recherche. L’igname, Plante Séculaire et Culture D’avenir, Cirad, Inra, Orstom, Coraf, CollColloques.

[4] FAOSTAT. http://www.fao.org/faostat/en/#data/QC/visualize.2019.

[5] Coursey D.G., Simmonds N.W. (1976). Yams. Evolution of Crops Plants.

[6] Azeteh I.N., Hanna R., Sakwe P.N., Njukeng A.P., Kumar P.L. (2019). Yam (*Dioscorea* spp.) production trends in Cameroon: A review. Afr. J. Agric. Res..

[7] Trèche S. (1989). Potentialités Nutritionnelles des Ignames (Dioscorea spp.) Cultivées au Cameroun.

[8] Lape I.M., Treche S. (1994). Nutritional quality of yams (*Dioscorea rotundata* and *Dioscorea dumetorum*) flours for growing rats. J. Sci. Food Agric..

[9] Dumont R., Hamon P., Seignobos C. (1994). Les Ignames au Cameroun.

[10] Hamon P., Dumon R., Zoundjihékpon J., Tio-Touré B., Hamon S. (1995). Wild yams in West Africa: Morphological characteristics. Les Ignames Sauvages d’Afrique de L’ouest: Caracteristiques Morphologiques.

[11] Iwu M.M., Okundji C.O., Ohiaeri G.O., Okah P., Corley D., Tempesta M.S. (1990). Hypoglycaemie activity of dioscoretine from tubers of *D. dumetorum* in normal and alloxan diabetic rabbits. Planta Med..

[12] Adegbite A.A., Adesiyan S.O., Agbaje G.O., Omoloye A.A. (2005). Host suitability of crops under yam intercrop to root-knot Nematode (*Meloidogyne incognita* Race 2) in South-western Nigeria. J. Agric. Rural Develop. Trop. Subtrop..

[13] Trèche S., Delpeuch F., Miege J., Lyonga S.N. (1982). Le durcissement de Dioscorea dumetorum au Cameroun. Yams-Ignames.

[14] Sonibare A.M., Asiedu R., Dirk C., Albach A. (2010). Genetic diversity of *Dioscorea dumetorum* (Knuth) Pax using Amplified Fragment Length Polymorphisms (AFLP) and cpDNA. Biochem. Syst. Ecol..

[15] Sartie A., Asiedu R., Franco J. (2012). Genetic and phenotypic diversity in a germplasm working collection of cultivated tropical yams (*Dioscorea* spp.). Genet. Resour. Crop Evol..

[16] Ngo Ngwe M.F.S., Joly S., Bourge M., Brown S., Omokolo D.N. (2014). Analysis of four cultivated species of yams (*Dioscorea* spp.) from Cameroon. J. Plant Breed. Genet..

[17] Ngo Ngwe M.F.S., Omokolo D.N., Joly S. (2015). Evolution and phylogenetic diversity of yam species (*Dioscorea* spp.): Implication for conservation and agricultural practices. PLoS ONE.

[18] Siadjeu C., Mahbou S.T.G., Bell J.M., Nkwate S. (2015). Genetic diversity of sweet yam “*Dioscorea dumetorum*” (Kunth) pax revealed by morphological traits in two agroecological zones of Cameroon. Afr. J. Biotechnol..

[19] Siadjeu C., Mayland-Quellhorst E., Albach D.C. (2018). Genetic diversity and population structure of trifoliate yam (*Dioscorea dumetorum* Kunth) in Cameroon revealed by genotyping-by-sequencing (GBS). BMC Plant Biol..

[20] Siadjeu C., Pucker B., Viehöver P., Albach D.C., Weisshaar B. (2020). High contiguity de novo genome sequence assembly of trifoliate yam (*Dioscorea dumetorum*) using long read sequencing. Genes.

[21] Yolou M., Zoundjihekpon J., Assaba E.I., Anizehou I., Akoegninnou A. (2015). La floraison des ignames africaines cultivées (*D. cayenensis*—*D. rotundata* et *D. dumetorum*) dans les champs des producteurs du Centre-Bénin. J. Appl. Biosci..

[22] Sadik S. A review of sexual propagation for yam improvement. Proceedings of the Fourth Symposium of the International Society for Tropical Root Crops, 1DRC-080e.

[23] Abraham K., Nair S.G., Sreekumri M.T., Unnikrishnan M. (1986). Seed set and seedling variation in greater yam (*Dioscorea alata* L.). Euphytica.

[24] Abraham K.P., Gopinathan N. (1990). Floral biology and artificial pollination in *Dioscorea alata* L.. Euphytica.

[25] Kafer J., Marais G.A.B., Pannell J.R. (2017). On the rarity of dioecy in flowering plants. Mol. Ecol..

[26] Walas Ł., Mandryk W., Thomas P.A., Tyrała-Wierucka Z., Iszkuło G. (2018). Sexual systems in gymnosperms: A review. Basic Appl. Ecol..

[27] Renner S.S., Ricklefs E.R. (1995). Dioecy and its correlates in the flowering plants. Am. J. Bot..

[28] Renner S.S. (2014). The relative and absolute frequencies of angiosperm sexual systems: Dioecy, monoecy, gynodioecy, and an updated online database. Am. J. Bot..

[29] Ming R., Bendahmane A., Renner S.S. (2011). Sex chromosomes in land plants. Annu. Rev. Plant Biol..

[30] Muyle A., Martin H., Zemp N., Mollion M., Gallina S., Tavares R., Silva A., Bataillon T., Widmer A., Glémin S. (2018). Dioecy in plants: An evolutionary dead end? Insights from a population genomics study in the *Silene* genus. bioRxiv.

[31] Montalvão A.P.L., Kersten B., Fladung M., Müller N.A. (2021). The diversity and dynamics of sex determination in dioecious plants. Front. Plant Sci..

[32] Bennett M.D., Grant W.F. (1984). The genome, the natural karyotype and biosystematics. Plant Biosystematics.

[33] Szinay D., Bai Y., Visser R., de Jong H. (2010). FISH applications for genomics and plant breeding strategies in tomato and other solanaceous crops. Cytogenet. Genome Res..

[34] Kwiatek M.T., Kurasiak-Popowska D., Mikołajczyk S., Niemann J., Tomkowiak A., Weigt D., Nawracala J. (2019). Cytological markers used for identification and transfer of *Aegilops* spp. chromatin carrying valuable genes into cultivated forms of *Triticum*. Comp. Cytogenet..

[35] Siljak-Yakovlev S., Benmalek S., Cerbah M., Bounaga N., Coba De La Pena T., Brown S.C., Sarr A. (1996). Chromosomal sex determination and heterochromatin structure in date palm. Sex Plant Reprod..

[36] Smith B.W. (1937). Notes on the cytology and distribution of the Dioscoreaceae. Bull. Torrey Bot. Club..

[37] Miège J. (1952). Contribution À L’Étude Systématique des *Dioscorea* D´Afrique Occidentale. Ph.D. Thesis.

[38] Miège J. (1954). Nombre chromosomique et répartition géographique de quelques plantes tropicales et équatoriales. Rév. Cytol. Biol. Vég..

[39] Martin F.W., Ortiz S. (1963). Chromosome numbers in some *Dioscorea* species. Cytologia.

[40] Baquar S.R. (1980). Chromosome behavior in Nigerian yams *(Dioscorea)*. Genetica.

[41] Araki H., Harada T., Yakuwa T. (1983). Some characteristics of interspecific hybrids between *Dioscorea japonica* Thumb. and *Dioscorea opposite* Thumb. J. Jpn. Soc. Hortic. Sci..

[42] Essad S., Maunoury C. (1984). Variation géographique des nombres chromosomiques de base et polyploïdie dans le genre *Dioscorea* à propos du dénombrement des espèces *transversa* Brown, *pilosiuscuia* Bert. et *trifida* L.. Agronomie.

[43] Zoundjihèkpon J., Essad S., Toure B. (1990). Dénombrement chomosomique dans dix groups variétaux du compexe *Dioscorea cayenensis-rotundata*. Cytologia.

[44] Rice A., Glick L., Abadi C. (2015). The Chromosome Counts Database (CCDB)—A community resource of plant chromosome numbers. New Phytol..

[45] Rama-Rao V., Murty O.R. (1975). Meiotic studies in species and hybrids in medicinal yams. Curr. Contents.

[46] Ramachandran K. (1968). Cytological studies in Dioscoreaceae. Cytologia.

[47] Takeuchi Y., Iwao T., Akahorl A. (1970). Chromosome numbers of some Japanese *Dioscorea* species. Acta Phytotax. Geobot..

[48] Ching H., Chang M., Ling P., Ting C., Doy F.A. (1985). cytotaxonomic study on Chinese *Dioscorea* L. the chromosome numbers and their relation to the origin and evolution of the genus. Acta Phytotax. Sin..

[49] Arumuganathan K., Earle E.D. (1991). Nuclear DNA content of some important plant species. Plant Mol. Biol. Rep..

[50] Dansi A., Daïnou O., Agbangla C., Ahanhanzo C., Brown S., Adoukonou-Sagbadja H. (2005). Ploidy level and nuclear DNA content of some accessions of water yam (*Dioscorea alata*) collected at Savè, a district of central Benin. Plant Genet. Resour. Newsl..

[51] Hamon P., Brizard J.P., Zoundjihékpon J., Duperray C., Borgel A. (1992). Etude des index d’ADN de huit ignames (*Dioscorea* spp.) par cytométrie en flux. Can. J. Bot..

[52] Scarcelli N., Dainou O., Agbangla C., Tostain S., Pham J.L. (2005). Segregation Patterns of izozyme loci and microsatellite markers show the diploidy of African yam *Dioscorea rotundata* (2n = 40). Theor. Appl. Genet..

[53] Arnau G., Nemorin A., Maledon E., Abraham K. (2009). Revision of ploidy status of *Dioscorea alata* (*Dioscoreaceae*) by cytogenetic and microsatellite segregation analysis. Theor. Appl. Genet..

[54] Obidiegwu J.E., Rodriguez E., Ene-obong E.E., Loureiro J., Muoneke C., Santos C., Kolesnikova-Allen M., Asiedu R. (2009). Estimation of the nuclear DNA content in some representatives of genus *Dioscorea*. Sci. Res. Essays.

[55] Leitch I.J., Chase M.W., Bennett M.D. (1998). Phylogenetic analysis of DNA C-values provides evidence for a small ancestral genome size in flowering plants. Ann. Bot..

[56] Ramachandran K. (1962). Studies on the cytology and sex determination of the Dioscoreaceae. J. Indian Bot. Soc..

[57] Bhat B.K., Bindroo B. (1980). Sex chromosomes in *Dioscorea deltoidea* wall. Cytologia.

[58] Martin F.W. (1966). Sex ratio and sex determination in *Dioscorea*. J. Hered..

[59] Terauchi R., Kahl G. (1999). Mapping of *Dioscorea tokoro* genome: AFLP markers linked to sex. Genome.

[60] Tamiru M., Natsume S., Takagi H., White B., Yaegashi H., Shimizu M., Yoshida K., Uemura A., Oikawa K., Abe A. (2017). Genome sequencing of the staple food crop white Guinea yam enables the development of a molecular marker for sex determination. BMC Biol..

[61] Cormier F., Lawac F., Maledon E., Gravillon M.C., Nudol E., Mournet P., Vignes H., Chaïr H., Arnau G. (2019). A reference high-density genetic map of greater yam (*Dioscorea alata* L.). Theor. Appl. Genet..

[62] Mondo J.M., Agre P.A., Asiedu R., Akoroda M.O., Asfaw A. (2021). Genome-Wide Association Studies for Sex Determination and Cross- Compatibility in Water Yam (Dioscorea alata L.). Plants.

[63] Terauchi R., Terachi T., Miyashita N.T. (1997). DNA polymorphism at the Pgi locus of a wild yam, *Dioscorea tokoro*. Genetics.

[64] Ngo Ngwe M.F.S. (2016). Evaluation de la Diversité Génétique des Dioscorea Spp. du Cameroun Et Développement des Techniques de Conservation.

[65] Ngo Ngwe M.F.S., Siljak-Yakovlev S. (2019). Heterochromatin and rDNA pattern revealed heteromorphic sex chromosomes in *Dioscorea dumetorum*. 1st Congress of Geneticists in Bosnia and Herzegovina. Genet. Appl..

[66] Lan T., Zhang S., Liu B., Li X., Chen R., Song W. (2006). Differentiating sex chromosomes of the dioecious *Spinacia oleracea* L. (spinach) by FISH of 45S rDNA. Cytogenet. Genome Res..

[67] Fujisawa M., Nakayama S., Nishio T., Fujichita M., Hayashi K., Ishizaki K., Kajikawa M., Yamato T.K., Fukuzawa H., Ohyama K. (2003). Evolution of ribosomal DNA unit on the X chromosome independent of autosomal units in the liverwort *Marchantia polymorpha*. Chromosome Res..

[68] Reed K.M., Phillips R.B. (1997). Polymorphism of the nucleolus organizer region (NOR) on the putative sex chromosomes of Arctic char (*Salvelinus alpinus*) is not sex related. Chromosome Res..

[69] Ocalewicz K., Woznicki P., Furgala-Selezniow G., Jankun M. (2011). Chromosomal location of Ag/CMA3 -NORs, 5S rDNA and telomeric repeats in two stickleback species. Ital. J. Zool..

[70] Proskuryakova A.A., Kulemzina A.I., Perelman P.L., Serdukova N.A., Ryder O.A., Graphodatsky A.S. (2018). The case of X and Y localization of nucleolus organizer regions (NORs) in *Tragulus javanicus* (Cetartiodactyla, Mammalia). Genes.

[71] Charlesworth D. (2002). Plant sex determination and sex chromosomes. Heredity.

[72] Hobza R., Kubat Z., Cegan R., Jesionek W., Vyskot B., Kejnovsky E. (2015). Impact of repetitive DNA on sex chromosome evolution in plants. Chromosome Res..

[73] Hobza R., Cegan R., Jesionek W., Kejnovsky E., Vyskot B., Kubat Z. (2017). Impact of repetitive elements on the y chromosome formation in plants. Genes.

[74] Bourge M., Brown S.C., Siljak-Yakovlev S. (2018). Flow cytometry as tool in plant sciences, with emphasis on genome size and ploidy level assessment. Genet. Appl..

[75] Lepers-Andrzejewski S., Siljak-Yakovlev S., Brown S.C., Wong M., Dron M. (2011). Diversity and dynamics of plant genome size: An example of polysomaty from a cytogenetic study of *Tahitian vanilla* (*Vanilla* x *tahitensis*, Orchidaceae). Am. J. Bot..

[76] Schweizer D. (1976). Reverse fluorescent chromosome banding with chromomycin and DAPI. Chromosoma.

[77] Siljak-Yakovlev S., Cerbah M., Coulaud J., Stoian V., Brown S.C., Zoldos V., Jelenic S. (2002). Nuclear DNA content, base composition, heterochromatin and rDNA in *Picea omorika* and *Picea abies*. Theor. Appl. Genet..

[78] Heslop-Harrison J.S., Schwarzacher T., Anamthawat-Jonsson K., Leitch I.J. (1991). In situ hybridization with automated chromosome denaturation techniques. J. Methods Cell Mol. Biol..

